# Supplementary material for: Introduction of loxP sites by electroporation in the mouse genome; a simple approach for conditional allele generation in complex targeting loci
Source: BMC Biotechnol. 2022 May 12;22:14. doi: 10.1186/s12896-022-00744-8 (PMC9097428; doi:10.1186/s12896-022-00744-8)
Supplement: Supplementary file 1 — Additional file 1. Details of the projects that were not completed using the Easi-CRISPR procedure. Table highlighting the details of the projects that were not completed using the Easi-CRISPR procedure. [file 12896_2022_744_MOESM1_ESM.docx]

**Additional file 1: Details of the projects that were not completed using the *Easi*-CRISPR procedure.**

| Gene name | Procedure | Number of embryos microinjected | Number of embryos that survived | Number of embryos implanted | Concentration (ng/μl) | Number of surgeries | Number of gestations | Number of pups born | Number of properly targeted pups | Random integration | Partial integration |
| --- | --- | --- | --- | --- | --- | --- | --- | --- | --- | --- | --- |
| *Sar1b* | *Easi*-CRISPR | 413 | 334 | 297 | 10:10:10-15:15:10 | 11 | 7 | 18 | 0 | 3 | 1 |
| *Loxl1* | *Easi*-CRISPR | 593 | 487 | 448 | 5:5:5-20:20:20 | 17 | 12 | 25 | 0 | 2 | 2 |
