# Supplementary material for: Introduction of loxP sites by electroporation in the mouse genome; a simple approach for conditional allele generation in complex targeting loci
Source: BMC Biotechnol. 2022 May 12;22:14. doi: 10.1186/s12896-022-00744-8 (PMC9097428; doi:10.1186/s12896-022-00744-8)
Supplement: Supplementary file 5 — Additional file 5. Repair template details for all eight projects. Table highlighting the repair template details for all eight projects. [file 12896_2022_744_MOESM5_ESM.docx]

**Additional file 5: Repair template details for all eight projects.**

| Gene name | Repair template name | Repair template sequence (5'-3') | Strand |
| --- | --- | --- | --- |
| *Icam1* | 892_loxP_Up | ATAAATGAATAAAAAGAAAGAAAAAGGGTGGTGTGTGGGGTGGACCCCAGAGGTCCAACTGAATTCATAACTTCGTATAAaGTATcCTATACGAAGTTATCATACTGAGTGGCAGCCTCCAGGATCACAAACAACACTTCTTTTGTTCTGCGGCCCTGGA | + |
| *Icam1* | 892_loxP_Dn | CAAAGATGGGCCAACCTGTCTCCTGAATGCTAGGACTAAATGACAAAGCCACTGCCATGTGCTAGCATAACTTCGTATAAaGTATcCTATACGAAGTTATCTAGTAAAATCTACGTTAGATAGACAGGGTTTCCCAGTGTAGATCAGGATGGCCTTGAAC | + |
| *Lox* | 901_loxP_Up | gccggtcagtgctGTCTAGACGTGGCGGCCTGGGCGCGGCGGCGGGATCCGGTCCCGCAGGAATTCataacttcgtataatgtatgctatacgaagttatACTCAACAACGCCCCCCCTCCCCAACCCCCAACCCCAGCCTGTCCCGCCAGCTCTGGAAT | - |
| *Lox* | 901_loxP_Dn | GGTATCTTGGGAAAGAACCTGAGACCTAGTCACCAGAATAAGGGTTTTCTTTTCCCTCCCataacttcgtatagcatacattatacgaagttatGCTAGCCGGGTATGCGACTTTCACTGAAGCAGCACGCATAAAAACATTCCAAATTAACTCTCTCTC | + |
| *Sar1b* | 911_loxP_Up1 | GAAATTTAATGGCATTTTGAGATTTATGCATAAGCATAGATGAGGTTCTATCAGCCTGGGataacttcgtataatgtatgctatacgaagttatTAACAGGGACTTGTTGTTTATGTATGCACACATGCATATGTTTATGTGCACCACAGTGTA | + |
| *Sar1b* | 911_loxP_Dn3 | TCAGTTGAGAAAGGCTTCACCTCTCCATACAGTAGACATGCTGACCAGAACCAACCTTAGataacttcgtatagcatacattatacgaagttatTTCCTAATTACTCGAGGTCTCAGATCACAGCTTCAGCCTTGTGAGGGCTGAGGTACAGGG | - |
| *Loxl1* | 902_loxP_Up1 | CCAGCTCCTGGGTCTTGTTCTCATTCCAAATCTTCTAGGCTGTCTCAATGGCTCCCACACataacttcgtataatgtatgctatacgaagttatCACCAAGCTGTAGAGGCAGTGAATTGCTACTAACCTCCTTGAGCCCAGGACCCTCTGACC | - |
| *Loxl1* | 902_loxP_Dn1 | TCACCCATGCCCAGCCATTCCCTGCTATGATCAGACAGGCTGCCAGGACCACACCCGTCCataacttcgtatagcatacattatacgaagttatCTGTCCACGCCCTACAGTTCTGTGCCCACTAGGACTTCCCCAACACCCACACTGACCACT | + |
| *Loxl1* | 902_loxP_Dn1_V2_NT | AGTGGTCAGTGTGGGTGTTGGGGAAGTCCTAGTGGGCACAGAACTGTAGGGCGTGGACAGataacttcgtataatgtatgctatacgaagttatGGACGGGTGTGGTCCTGGCAGCCTGTCTGATCATAGCAGGGAATGGCTGGGCATGGGTGA | - |
| *Pard6a* | 938_loxP_Up1 | CTCCATCCTCCGTCCTATTCTGCTTTTCTGTCTGCCTCCCCTCATCGCATCCTCCCTCCCataacttcgtataatgtatgctatacgaagttatACTACGGACTTCCAGAGTCCCTCTGTGGCCTGGGTATGTGGAGAGCAAGGGAAAGACCTT | + |
| *Pard6a* | 938_loxP_Up2 | CATCCTCCGTCCTATTCTGCTTTTCTGTCTGCCTCCCCTCATCGCATCCTCCCTCCCACTataacttcgtataatgtatgctatacgaagttatACGGACTTCCAGAGTCCCTCTGTGGCCTGGGTATGTGGAGAGCAAGGGAAAGACCTTGGA | + |
| *Pard6a* | 938_loxP_Dn1 | GCTACTTGGAGGTCAGACCAAGGGCAGAGGCTACAGAACATAAAGCCTGGGGGCCCTGGGataacttcgtatagcatacattatacgaagttatTATGATCTACCCTGATATACCTGTCCTATCACCCTAACCCAAGAAAGCTGCCCACTGTAC | - |
| *Pard6a* | 938_loxP_Dn2 | ATATCAGGGTAGATCATACCCAGGGCCCCCAGGCTTTATGTTCTGTAGCCTCTGCCCTTGataacttcgtataatgtatgctatacgaagttatGTCTGACCTCCAAGTAGCAGGAAAACAGTTGACATTGTCCCTATCACAGGCCCAATTGGG | + |
| *Pard6g* | 939_loxP_Up1 | AAGCAATGGTGAGGACAAGGCCTCCAGGTCTGCTCCACTTGCCTGCCTCAGTTGCCAGGTataacttcgtatagcatacattatacgaagttatTGTAGGTGGTCTCGCCCATGAAGTAGTGAACTCAGGCAGGACGGAAAGCGACAGGATAGT | - |
| *Pard6g* | 939_loxP_Up3 | AACTATTTCTTTACAATTTGGCATCGGTTTACTATCCTGTCGCTTTCCGTCCTGCCTGAGataacttcgtataatgtatgctatacgaagttatTTCACTACTTCATGGGCGAGACCACCTACAACCTGGCAACTGAGGCAGGCAAGTGGAGCA | + |
| *Pard6g* | 939_loxP_Dn1_V2 | TAAAAGGCTCCAGGGACTTCTCCCACCCGACCCTAAGCAACAGCAGCTCCCCATCCCGCAataacttcgtatagcatacattatacgaagttatGGGTCCGCGTTGCAGACCCTCCCAGACCAAAGCTTTTGCAGCGAAGGAGCTGCCTCCGCA | - |
| *Pard6g* | 939_loxP_Dn2 | AGTCAGTAAAAGGCTCCAGGGACTTCTCCCACCCGACCCTAAGCAACAGCAGCTCCCCATataacttcgtatagcatacattatacgaagttatCCCGCAGGGTCCGCGTTGCAGACCCTCCCAGACCAAAGCTTTTGCAGCGAAGGAGCTGCC | - |
| *Clcf1* | 874_loxP_Up | CCAGCCAGAGGAGAGTCCATATCAAAGGCAAAGGGACCTGCTGACACCAAAACTCCTCCTataacttcgtatagcatacattatacgaagttatCAACAGGCCAAAGGGACAAGAGATAGCAATGTACTTACCCCAAAGAAGAGAACTCAGTTC | - |
| *Clcf1* | 874_loxP_Dn | TCTGGCTTGCTAACTACTAAGCCCTGACATTCAGTGCCTAGCTTCCCCAGGCCTCCATTAataacttcgtataatgtatgctatacgaagttatGTCCCATCAGGGGCCCTCACCTTCCTCTTCTGGAAGGCTGACAGACAATAGCCAAGCTAG | + |
| *Mapkapk5* | 933_loxP_Up | CAGTACCTGTCACAGCACAGGTAAAGGAGTGGCCAGGCATCTTACAGGATGTGTCCACCGataacttcgtataatgtatgctatacgaagttatCTTTATTCTGACAGAGGATCATTTGTAGTCGTGCTTGTTCAGTAAATATAGGTTATAGTT | - |
| *Mapkapk5* | 933_loxP_Dn | GTTCAGTTAGAGTTGCTGTTAGCCCTGATATAGGCTGTGTTCTTTTCCTGGTTTCCTGTCataacttcgtataatgtatgctatacgaagttatCATATTCAGTATACTAGTCAGTTGCTCCAGTTGCCAGTCTTCAGAGTGAGGCTGGGTGGA | - |
