# Supplementary material for: Introduction of loxP sites by electroporation in the mouse genome; a simple approach for conditional allele generation in complex targeting loci
Source: BMC Biotechnol. 2022 May 12;22:14. doi: 10.1186/s12896-022-00744-8 (PMC9097428; doi:10.1186/s12896-022-00744-8)
Supplement: Supplementary file 6 — Additional file 6. Guide sequence details for all eight projects. Table highlighting the guide sequence details for all eight projects. [file 12896_2022_744_MOESM6_ESM.docx]

**Additional file 6: Guide sequence details for all eight projects.**

| Gene name | Guide name | Guide sequence (5'-3' + PAM site) | Strand | Genome coordinate (GRCm38/mm10) |
| --- | --- | --- | --- | --- |
| *Icam1* | 892_crRNA_Up | GGCTGCCACTCAGTATGAGT TGG | - | Chr9:21,026,167-21,026,189 |
| *Icam1* | 892_crRNA_Dn | AACGTAGATTTTACTAGACA TGG | - | Chr9:21,029,092-21,029,114 |
| *Lox* | 901_crRNA_Up | GGGGGGCGTTGTTGAGTCTG CGG | + | Chr18:52,528,597-52,528,619 |
| *Lox* | 901_crRNA_Dn | TGAAAGTCGCATACCCGGGG AGG | - | Chr18:52,528,086-52,528,108 |
| *Sar1b* | 911_crRNA_Up1 | ACAACAAGTCCCTGTTACCC AGG | - | Chr11:51,777,174-51,777,196 |
| *Sar1b* | 911_crRNA_Dn3 | CCTCGAGTAATTAGGAACTA AGG | + | Chr11:51,777,781-51,777,803 |
| *Loxl1* | 902_crRNA_Up1 | GCCTCTACAGCTTGGTGGTG TGG | + | Chr9:58,297,872-58,297,894 |
| *Loxl1* | 902_crRNA_Dn1 | CTGTAGGGCGTGGACAGGGA CGG | - | Chr9:58,297,266-58,297,288 |
| *Pard6a* | 938_crRNA_Up1 | CTCTGGAAGTCCGTAGTGGG AGG | - | Chr8:105,701,959-105,701,981 |
| *Pard6a* | 938_crRNA_Up2 | GGACTCTGGAAGTCCGTAGT GGG | - | Chr8:105,701,962-105,701,984 |
| *Pard6a* | 938_crRNA_Dn1 | TATCAGGGTAGATCATACCC AGG | + | Chr8:105,702,451-105,702,473 |
| *Pard6a* | 938_crRNA_Dn2 | CTACTTGGAGGTCAGACCAA GGG | - | Chr8:105,702,504-105,702,526 |
| *Pard6g* | 939_crRNA_Up1 | GGGCGAGACCACCTACAACC TGG | + | Chr18:80,046,591-80,046,613 |
| *Pard6g* | 939_crRNA_Up3 | GCCCATGAAGTAGTGAACTC AGG | - | Chr18:80,046,572-80,046,594 |
| *Pard6g* | 939_crRNA_Dn1_V2 | GTCTGCAACGCGGACCCTGC GGG | + | Chr18:80,048,053-80,048,075 |
| *Pard6g* | 939_crRNA_Dn2 | AACGCGGACCCTGCGGGATG GGG | + | Chr18:80,048,059-80,048,081 |
| *Clcf1* | 874_crRNA_Up | GTCCCTTTGGCCTGTTGAGG AGG | + | Chr19:4,221,360-4,221,382 |
| *Clcf1* | 874_crRNA_Dn | AGGGCCCCTGATGGGACTAA TGG | - | Chr19:4,223,878-4,223,900 |
| *Mapkapk5* | 933_crRNA_Up1 | CCTCTGTCAGAATAAAGCGG TGG | + | Chr5:121,536,160-121,536,182 |
| *Mapkapk5* | 933_crRNA_Dn2 | CTAGTATACTGAATATGGAC AGG | + | Chr5:121,535,161-121,535,183 |
