# Supplementary material for: Introduction of loxP sites by electroporation in the mouse genome; a simple approach for conditional allele generation in complex targeting loci
Source: BMC Biotechnol. 2022 May 12;22:14. doi: 10.1186/s12896-022-00744-8 (PMC9097428; doi:10.1186/s12896-022-00744-8)
Supplement: Supplementary file 7 — Additional file 7. PCR oligo details for all eight projects. Table highlighting the PCR oligo details for all eight projects. [file 12896_2022_744_MOESM7_ESM.docx]

**Additional file 7: PCR oligo details for all eight projects.**

| Gene name | Oligo Name | Oligo Sequence (5' to 3') | Strand | Genome coordinate (GRCm38/mm10) |
| --- | --- | --- | --- | --- |
| *Icam1* | 892_Up_Fwd | GAGGAAGAAGACCCCAGCAAT | + | chr9:21,025,894-21,025,914 |
| *Icam1* | 892_loxP_Up_Fwd | CCAGAGGTCCAACTGAATTCATAACTTCGTATAAa | + | NA |
| *Icam1* | 892_Up_Rev | CTCCAGGATCTGGTCCGCTA | - | chr9:21,026,555-21,026,574 |
| *Icam1* | 892_Dn_Fwd | ACTCCTTAGAAGGGTCGTGC | + | chr9:21,028,716-21,028,735 |
| *Icam1* | 892_loxP_Dn_Rv | TCTAACGTAGATTTTACTAGATAACTTCGTATAGg | - | NA |
| *Icam1* | 892_Dn_Rev | CACAGTTCTGGACCTGCTCTT | - | chr9:21,029,268-21,029,288 |
| *Lox* | 901_Up_Fwd3 | AGCTGATGACCTCTGACTCCT | + | chr18:52,528,402-52,528,422 |
| *Lox* | 901_loxP_UP_Rv | ATCCGGTCCCGCAGGAATTCataacttcgtataat |  | NA |
| *Lox* | 901_Up_Rv3 | ACATAGATCGCATGGTGGGC | - | chr18:52,528,844-52,528,863 |
| *Lox* | 901_Dn_Fwd | ACACCGTGACGAATACACCC | + | chr18:52,527,891-52,527,910 |
| *Lox* | 901_loxP_Dn_Fwd | AGGGTTTTCTTTTCCCTCCCataacttcgtatagc |  | NA |
| *Lox* | 901_Dn_Rev | GTGGGCGAAAGGTGACAAAC | - | chr18:52,528,429-52,528,448 |
| *Sar1b* | 911_Up_Fwd | CATCACACATGGCTCTTCATTT | + | chr11:51,777,029-51,777,050 |
| *Sar1b* | 911_loxP_Up_Fwd | TGAGGTTCTATCAGCCTGGGataacttcgtataat |  | NA |
| *Sar1b* | 911_Up_Rev | TGATGTGCTCAGTTCAGTCCTT | - | chr11:51,777,296-51,777,317 |
| *Sar1b* | 911_LoxP_Up_Rv | TAAACAACAAGTCCCTGTTAataacttcgtatagc |  | NA |
| *Sar1b* | 911_Dn_Fwd | ACTTCGGCTTTGCTCTTTGTAG | + | chr11:51,777,565-51,777,586 |
| *Sar1b* | 911_LoxP_Dn_Fw | AGACCTCGAGTAATTAGGAAataacttcgtataat |  | NA |
| *Sar1b* | 911_Dn_Rev2 | CACCACTAACTCCTGAGCACTC | - | chr11:51,777,991-51,778,012 |
| *Sar1b* | 911_loxP_Dn_Rev | CTGACCAGAACCAACCTTAGataacttcgtatagc |  | NA |
| *Loxl1* | 902_up_Fw | ATGGGCTCTCTGCACGTATG | + | chr9:58,297,665-58,297,684 |
| *Loxl1* | 902_loxP_up_Fw | ACTGCCTCTACAGCTTGGTGataacttcgtatagc |  | NA |
| *Loxl1* | 902_up_Rv | ACGGCTCGCTATAGAGGTGA | - | chr9:58,298,101-58,298,120 |
| *Loxl1* | 902_loxP_Up_Rev | TGTCTCAATGGCTCCCACACataacttcgtataat |  | NA |
| *Loxl1* | 902_dn_Fw | TGTCCCCTTTAACCCGGTCT | + | chr9:58,297,082-58,297,101 |
| *Loxl1* | 902_loxP_Dn_Fwd | TGCCAGGACCACACCCGTCCataacttcgtatagc |  | NA |
| *Loxl1* | 902_dn_Rv | AGCAGAGTGCCTTGGCATAG | - | chr9:58,297,577-58,297,596 |
| *Loxl1* | 902_loxP_Dn_Rv | GAACTGTAGGGCGTGGACAGataacttcgtataat |  | NA |
| *Pard6a* | 938_Up_Fwd | TGAAGAGCAAAGTAAGGGCTTC | + | chr8:105,701,793-105,701,814 |
| *Pard6a* | 938_loxP_Up1_Fw | CTCATCGCATCCTCCCTCCCataacttcgtataat |  | NA |
| *Pard6a* | 938_loxP_Up2_Fw | ATCGCATCCTCCCTCCCACTataacttcgtataat |  | NA |
| *Pard6a* | 938_Up_Rev | GGCTTAAACCTCAGCTCCAGTA | - | chr8:105,702,040-105,702,061 |
| *Pard6a* | 938_LoxP_Up1_Rv | GGACTCTGGAAGTCCGTAGTataacttcgtatagc |  | NA |
| *Pard6a* | 938_LoxP_Up2_Rv | GAGGGACTCTGGAAGTCCGTataacttcgtatagc |  | NA |
| *Pard6a* | 938_Dn_Fwd | GTGTGTGGTACACCAGATCCCT | + | chr8:105,702,248-105,702,269 |
| *Pard6a* | 938_LoxP_Dn2_Fw | TTCTGTAGCCTCTGCCCTTGataacttcgtataat |  | NA |
| *Pard6a* | 938_Dn_Rev | TCTTGCGCCTTTGTAGAGAGTT | - | chr8:105,702,643-105,702,664 |
| *Pard6a* | 938_loxP_Dn1_Rev | TAAAGCCTGGGGGCCCTGGGataacttcgtatagc |  | NA |
| *Pard6a* | 938_loxP_Dn2_Rv | CTGCTACTTGGAGGTCAGACataacttcgtatagc |  | NA |
| *Pard6g* | 939_Up_Fw2 | TCCTCAAACCCTCCTGCAAGTG | + | chr18:80,046,428-80,046,449 |
| *Pard6g* | 939_loxP_up1_Fw | CATGGGCGAGACCACCTACAataacttcgtataat |  | NA |
| *Pard6g* | 939_loxP_up3_Fw | CGCTTTCCGTCCTGCCTGAGataacttcgtataat |  | NA |
| *Pard6g* | 939_Up_Rv | AGAAGCTCTGGGCCATTTTCT | - | chr18:80,046,734-80,046,754 |
| *Pard6g* | 939_loxP_up1_Rv | GCCTGCCTCAGTTGCCAGGTataacttcgtatagc |  | NA |
| *Pard6g* | 939_loxP_up3_Rv | CTCGCCCATGAAGTAGTGAAataacttcgtatagc |  | NA |
| *Pard6g* | 939_Dn_V2_Fwd | TAGACAAGACCACAATCCGCTA | + | chr18:80,047,843-80,047,864 |
| *Pard6g* | 939_loxP_Dn1V2_Fw | AGGGTCTGCAACGCGGACCCataacttcgtataat |  | NA |
| *Pard6g* | 939_loxP_Dn2V2_Fw | TGCAACGCGGACCCTGCGGGataacttcgtataat |  | NA |
| *Pard6g* | 939_Dn_Rv2 | AATGGATCTCAGGCCTTAGGCC | - | chr18:80,048,239-80,048,260 |
| *Pard6g* | 939_loxP_Dn1v2_Rv | CAGCAGCTCCCCATCCCGCAataacttcgtatagc |  | NA |
| *Pard6g* | 939_loxP_Dn2v2_Rv | AAGCAACAGCAGCTCCCCATataacttcgtatagc |  | NA |
| *Clcf1* | 874_v2_Up_Fwd_Long | CCCATGCCCTCCCTCCTGTCCTCTCTTTAGATTT | + | chr19:4,221,250-4,221,283 |
| *Clcf1* | 874_loxP_v2_Up_Fwd | CTTGTCCCTTTGGCCTGTTGataacttcgtataat |  | NA |
| *Clcf1* | 874_loxP_V2_up_Rv | CTGACACCAAAACTCCTCCTataacttcgtatagc |  | NA |
| *Clcf1* | 874_up1_Rv | CAGCTTGCCAGCAGAGAGATA | - | chr19:4,222,008-4,222,028 |
| *Clcf1* | 874_Dn_Fwd | ACCCTAACCCTACCTGCCAT | + | chr19:4,223,545-4,223,564 |
| *Clcf1* | 874_loxP_v2_Dn_Rev | GTGAGGGCCCCTGATGGGACataacttcgtatagc |  | NA |
| *Clcf1* | 874_Dn_Rev_long_Ext_HA | GATCGGGGTTTCCATTATCCCTCCCATCGA | - | chr19:4,224,012-4,224,041 |
| *Mapkapk5* | 933_Dn_Fwd | CCCCCGACATATACTTGTAAGC | + | chr5:121,535,049-121,535,070 |
| *Mapkapk5* | 933_loxP_Dn_Fw | TGACTAGTATACTGAATATGataacttcgtatagc |  | NA |
| *Mapkapk5* | 933_loxP_Dn_Rv | TCTTTTCCTGGTTTCCTGTCataacttcgtataat |  | NA |
| *Mapkapk5* | 933_Dn_Rev | ACTCTTTCCTTGTTAGGGCCTC | - | chr5:121,535,363-121,535,384 |
| *Mapkapk5* | 933_Up_Fwd | AAAGACTTGGGACACCAAGAAA | + | chr5:121,536,072-121,536,093 |
| *Mapkapk5* | 933_loxP_Up_Fw | GATCCTCTGTCAGAATAAAGataacttcgtatagc |  | NA |
| *Mapkapk5* | 933_loxP_Up_Rv | CTTACAGGATGTGTCCACCGataacttcgtataat |  | NA |
| *Mapkapk5* | 933_Up_Rv_Long | GACTAAAGGATGAGATGTGACCATCTGTAAAATAA | - | chr5:121,536,422-121,536,456 |
